# Sequence Analysis of Staphylococcus hyicus ATCC 11249^T^, an Etiological Agent of Exudative Epidermitis in Swine, Reveals a Type VII Secretion System Locus and a Novel 116-Kilobase Genomic Island Harboring Toxin-Encoding Genes

**DOI:** 10.1128/genomeA.01525-14

**Published:** 2015-02-19

**Authors:** Michael J. Calcutt, Mark F. Foecking, Hsin-Yeh Hsieh, Pamela R. F. Adkins, George C. Stewart, John R. Middleton

**Affiliations:** a Department of Veterinary Pathobiology, University of Missouri, Columbia, Missouri, USA; b Bond Life Science Center, University of Missouri, Columbia, Missouri, USA; c Department of Veterinary Medicine and Surgery, University of Missouri, Columbia, Missouri, USA

## Abstract

Staphylococcus hyicus is the primary etiological agent of exudative epidermitis in swine. Analysis of the complete genome sequence of the type strain revealed a locus encoding a type VII secretion system and a large chromosomal island harboring the genes encoding exfoliative toxin ExhA and an EDIN toxin homolog.

## GENOME ANNOUNCEMENT

Exudative epidermitis (EE) of swine, or “greasy pig disease,” is predominantly caused by toxigenic Staphylococcus hyicus, although lesions following Staphylococcus chromogenes, Staphylococcus sciuri, and Staphylococcus aureus infection have also been reported (1). Diseases associated with S. hyicus also occur in horses (2) and cattle (3, 4), but the true incidence of S. hyicus in bovine mastitis requires re-evaluation following the recent description of the phylogenetically closely related species Staphylococcus agnetis (5).

A primary virulence factor of S. hyicus is exfoliative toxin Exh, a serine protease that cleaves the desmosomal intercellular adhesion protein desmoglein1 (6). An IgG-binding protein has also been reported (7), but the complete array of surface proteins and toxins has not been comprehensively assessed. Accordingly, the genome sequence of the type strain (isolated in Israel in 1950 [8]) was determined and analyzed.

Using the Pacific Biosciences system at NCGR (Santa Fe, NM, USA) sequence reads from two SMRT cells were generated for S. hyicus ATCC 11249^T^ and assembled (HGAP version 2 [9]). Optical mapping of NcoI fragments (Opgen) confirmed the assembly. The complete genome contains 2,472,129 bp, is 35.58% G+C, and is covered at a 96-fold depth. Manual curation was performed on an auto-annotated genome (PGAP, NCBI), resulting in a 2,400-gene set encoding 19 rRNAs, 58 tRNAs, 2,278 coding sequences, and 40 verified pseudogenes.

The availability of draft genome sequences for the most closely related taxa S. chromogenes (10) and S. agnetis (11) enabled comparative analyses to identify regions of difference. The largest such region was a 116-kb genomic island in S. hyicus, bounded by a recombinase gene. Two additional recombinases are encoded within the island, possibly reflecting a composite structure assembled through multiple incursions. The locus is primarily populated by genes encoding hypothetical proteins and bacteriophage-related genes, but harbors a five-gene cluster encoding ExhA toxin (12), tandem glutamyl-endopeptidase paralogs, and an open reading frame with 64% identity to the epidermal cell differentiation inhibitor (EDIN) toxin (a RhoA-targeting ADP-ribosylating toxin) of S. aureus (13). Database queries revealed that this is the first identification of an EDIN-like toxin among staphylococci other than S. aureus. Furthermore, in at least one S. aureus lineage, these toxin genes reside in a 9-kb pathogenicity island (14). The features of the *exhA* gene island reported here indicate that these two S. hyicus toxins are encoded by a pathogenicity island or prophage-related element. An additional toxin homolog, delta hemolysin, is encoded within RNA III at a different genomic locale, as described for S. aureus (15) and other *Staphylococcaceae*.

Additional regions of difference included an 8-gene locus encoding a type VII secretion system with identical configuration to that in S. aureus (16) and a putative gas vesicle protein gene cluster, similar to that found in the same genomic location in S. agnetis (11) but absent from S. chromogenes.

The complete genome sequence presented here is the first for the species and expands our understanding of potential virulence determinants for EE and provides insight into evolution of a dermatosis pathogen through genetic island acquisition.

### Nucleotide sequence accession number.

This complete genome sequence has been deposited at DDBJ/EMBL/GenBank under the accession number CP008747.
